# Supplementary material for: A Global Screen for Assembly State Changes of the Mitotic Proteome by SEC-SWATH-MS
Source: Cell Syst. 2020 Feb 26;10(2):133–155.e6. doi: 10.1016/j.cels.2020.01.001 (PMC7042714; doi:10.1016/j.cels.2020.01.001)

Q8WV41 | SNX33_HUMAN | SNX33 SH3PX3 SH3PXD3C SNX30
Monomer MW [kDa]: 65.265 Monomer expected elution fraction: 44

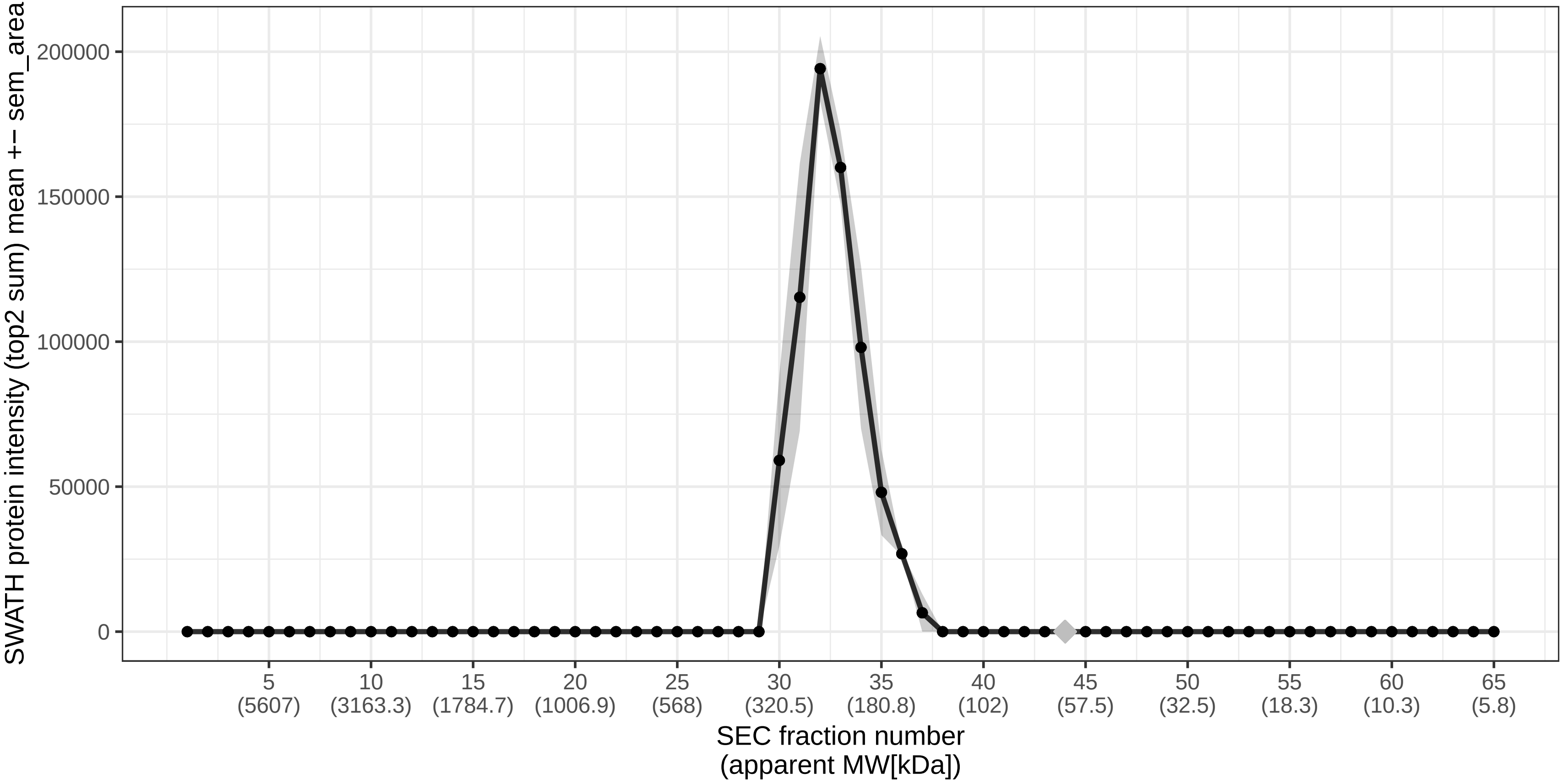

Supplement: Data S1. SEC-SWATH-MS Protein Chromatograms, Related to Figure 1 [file mmc6.zip › SECchrom_Q8WV41_SNX33_HUMAN_SNX33_SH3PX3_SH3PXD3C_SNX30.pdf]
